# Monthly Summary

**Published:** 1875-03

**Authors:** 


### MONTHLY SUMMARY.

---

*Supplemental Nerve Force.*—A surgeon of Lyon, M. E. Letevant, has demonstrated, in a work published in Paris, the following interesting propositions:—When a certain group of muscles, supplied by a particular nerve, are paralyzed as the result of a section of that nerve, the motions properly belonging to this group of muscles are not in all cases wholly abrogated, adjacent muscles, supplied by unaffected nerves, being capable of accomplishing some part of the actions, even though feebly. Similarly, when a sensitive nerve is divided, there is not total loss of sensibility experienced throughout the entire region of its anatomical distribution, the sensation being preserved, more or less feebly, either by means of the anastomosis of adjacent nerves, or by the conveyance of impressions indirectly to healthy neighboring cutaneous papillæ.—*Med. and Surg. Reporter.*

*False Tongue.*—Under the above caption the Visalia (Cal.) *Delta* says: "No doubt everybody thinks he knows what we mean by this heading. But what we do mean is a disease by that name, several cases of which have occurred on Tule River. The patient is taken with itching on the under side, at the root of the tongue, from which we are informed, commences a growth of a fungus resembling a tongue, which soon fills the mouth and protrudes from it, causing suffocation and death in a few days, unless relief is obtained.—*Med. and Surg. Reporter.*

---

*New Sign of Death.*—Dr. Monteverdi, of Cremona, recommends what he calls an "easy, prompt and certain" method of demonstrating the reality of death in man. It consists in the observation of a spot produced by the injection of a drop or two of ammonia beneath the skin. If the man be living, the spot produced by such injection is always of more or less vinous red, whilst, if he be dead, the color is scarcely different from that of the skin itself, or at least, has no purple tint. The solution of ammonia injected should have a strong odor of the gas, and a specific gravity of 0-92. If the individual be living, no bad effects are produced, beyond a burning pain, which lasts for a short time, and the reddish tint appears almost immediately. The spot appears to be due to the action of the ammonia on the blood.—*Med. and Surg. Reporter.*

---

*Treatment of Epistaxis.*—Dr. W. I. Wilson, U. S. A., reports the successful employment, in two cases of severe epistaxis, of a solution of the liquor ferri persulphatis, one part to three of water, introduced into the nostril in the form of spray, by means of the ether-spray apparatus. The bleeding was checked in a few seconds.—*Med. Times.*

---

*Danger of Chloroform After Chloral.*—From a late discussion in the Surgical Society of Paris we find that several members have been much alarmed at the profound stupor which seized patients who, after taking syrup of chloral, are made to inhale chloroform.—*Lancet.*

---

*An Insecticide.*—As the best of insecticides, a German chemist recommends a tincture of nux vomica prepared with caustic solution of ammonia. Bedbugs, cock-roaches, etc., are at once destroyed by it, and it is said that if a horse's harness is painted with it, the flies will avoid him! Against ants, castor oil is found an entire protection.

*On the Preservation of Leeches.*—Many pharmacists have no doubt, found the preservation of leeches a matter of great annoyance and loss.

The more common usage is to place them in earthen jars with perforated covers, and containing a quantity of earth; the leeches looked to occasionally to ascertain their sanitary condition.

I have also known the druggist to place old water-logged sticks, with a few pebbles, in a proper vessel, forming a sort of artificial pond. The leeches free themselves from the slime which collects upon them by drawing themselves through the debris at the bottom of the aquarium.

The following method for the preservation of leeches, has, in my experience, been most successful. Half a dozen "tenpenny" nails are placed at the bottom of a glass jar holding about eight pints—upon them is placed a piece of very porous sponge.

The jar being filled with water, we have an aquarium in which two dozen leeches will live for many months without a single loss by death; a piece of lawn of about one hundred meshes to the square inch should be secured by a strong rubber band to the top of the jar in order to prevent the escape of the leeches.

When the water is changed, the sponge should be thoroughly cleansed, to remove a quantity of slimy matter from the cells.

It said that the presence of metallic iron in water prevents it from becoming putrid. This influence is very marked in water in which leeches are preserved, in consequence of which, the change of the water becomes unnecessary except after long intervals.

The jar containing the leeches should be kept at a comparative temperature of from 55° to 66° F., excluded from sunlight, and a quantity of fresh water added every five or six days.—*The Laboratory.*

---

*Chloral as a Preservative.*—"Among the bodies preserved we were shown one, that of a middle-aged woman, injected in February last, and which is still in a perfect state of preservation, without any sign of decay or any trace of an offensive odor. The body looks as if it had been embalmed; that is, it resembles a body in the earliest stage of mummification, and on inquiring how this was effected, we were informed that it was by injecting into the body a solution of the hydrate of chloral in the proportion of one to ten parts. A mixture of carbolic acid and glycerine in the same proportions is sometimes used, but this is not found so efficacious as the chloral."—*London Medical Times and Gazette.*

*New Operation for Certain Cases of Cleft Palate and Blind Uvula.*—He remarked: At the present time, the operation for closing the cleft in the soft palate, as revived by Roux, and practiced by Dieffenbach, Warren, and others, and materially improved by Sir W. Fergusson, consists in dissecting off the margins of the fissur, bringing the edgee together, and holding them in apposition by suture, and in dividing the tensor and levator palati, and the palato-pharyngei muscles, in order to relieve tension, and place the parts as much at rest as possible, until union takes place, when the sutures sre to be removed.

There are frequently cases met with, however, in which each half of the split uvula and velum is large and thick, and in which the cleft does not extend entirely through the velum up to the hard palate. In such cases I have to recommend a different mode of procedure, one in which no suture is used, and with which, in the two cases I have had, I succeeded perfectly well.

The first case was in May last, in a boy, N. Y., aged ten years. He came from Western Virginia; was healthy in every respect, and allowed me to touch his palate without any discomfort to himself. The uvula was thick, and the whole palate was large, the cleft extending a little above, I thought about a fourth of the way through the velum. I had hardly finished my examination before I thought it possible to practice on this palate what I had seen Nelaton perform in a case of hare-lip, namely, to make an incision from a little below the middle of the right half of the uvula, on one side, and carrying the bistoury up to a point above the arch, of the fissure, and then turning the bistoury, to bring it down to a corresponding point in the uvula of the opposite side.

The column of flesh on either side of the fissure, now liberated by this incision, turns on its attached pedicle, or falls from the arch, leaving a long uvula with an oval opening in its base.

This operation having been practiced in the case referred to, in a few days granulations sprang up, and the parts contracted. A day or two later, when the upper part of the wound began to widen, I divided with a double-edged knife the tensor and levator palatia muscles, as recommended by Sir W. Fergusson, thus removing all tension, when the upper edges of the trapezoidal-shaped opening approached the median line; and under the application of a little nitrate of silver, cicatrization and narrowing of the entire opening rapidly ensued.

---

*Malignant Pustule Produced by a Fly-bite.*—The patient, a coachman, was conscious of being bitten by a fly on the 28th of March last. The puncture was inflicted in the temporal region, and a minute mark was left by it, not unlike a flea-bite, at which point some itching was experienced upon the following day. Upon the evening of the 20th, a swelling appeared in the temporal region, which extended over the entire face, becoming so intense that, upon the evening of the 30th, the eye-lids could no longer be parted. April 1st, he was received into the hospital, upon which occasion the characteristic black eschar was noticed surrounded by a coronet of vesicles. In spite of the most heroic treatment [incisions and cauterization,] no decided relief was obtained, the swelling and gangrene slowly extending until the left eyelids, the soft tissues of the nose, the lips and the left ear all sloughed and became detached, imparting to the face the appearance of a hideous mask. Death finally ensued May 10th. No trace of bacteria could be detected either in the blood, nor the serum of the vesicles, and the inoculation of rabbits with the blood was followed by negative results.—*Lyon Medical.*

---

*Lock-jaw and Quinia.*—Several cases of tetanus have followed hypodermic injections of sulphate of quinia, which will render medical men more careful in thus employing it. M. Bourdon says the following preparation is not irritating, and may be injected without danger: By weight, sulphate of quinia two parts, tartaric acid one part, water forty parts; mix.—*Scientific American.*

---

*A Patent Decision Affecting Dentistry.*—For several years past the dockets of all the United States Circuit Courts have been crowded with suits brought by the Goodyear Vulcanite Company against dentists, for infringing the Cummings patent, covering the hard rubber plates used for inserting artificial teeth. Between 2,900 and 3,000 suits have been brought against infringers of this patent. Judge Emmons, in behalf of the U. S. Court, of Michigan, has just rendered an opinion fully sustaining the validity of the patent.—*Med. Rec.*

---

*Precautions Against Trichina.*—The Medical Society of Kalamazoo, Mich., gives the following advice: Eat no uncooked or half-cooked hog's flesh. The raw flesh of a hog, whatever its shape or condition, whether ham, bacon or pork, salt or fresh, smoked or un-smoked, is liable to contain this parasite, full of life and activity, that may work a remediless mischief in the human body. Bologna sausage, if pork be in it uncooked, is as dangerous as any form of this meat. The heat that cooks meat utterly destroys the life and mischievous power of these vermin, and no one need fear any harm if this fact is observed.—*Med. and Surg. Reporter.*

